# Adolescentes: comportamento e risco cardiovascular

**DOI:** 10.1590/1677-5449.011816

**Published:** 2017

**Authors:** Ivelise Fhrideraid Alves Furtado da Costa, Carla Campos Muniz Medeiros, Fernanda Dayenne Alves Furtado da Costa, Camilla Ribeiro Lima de Farias, Diogo Rodrigues Souza, Wellington Sabino Adriano, Mônica Oliveira da Silva Simões, Danielle Franklin Carvalho

**Affiliations:** 1 Universidade de Pernambuco – UPE, Faculdade de Enfermagem Nossa Senhora das Graças, Recife, PE, Brasil.; 2 Universidade Estadual da Paraíba – UEPB, Programa de Mestrado em Saúde Pública, Campina Grande, PB, Brasil.; 3 Universidade Federal de Campina Grande – UFCG, Centro de Educação e Saúde, Campina Grande, PB, Brasil.; 4 Centro Universitário Unifacisa, Departamento de Enfermagem, Campina Grande, PB, Brasil.

**Keywords:** atividade física, comportamento sedentário, doenças cardiovasculares, adolescentes

## Abstract

**Contexto:**

Os benefícios para a saúde decorrentes da prática regular de atividade física estão bem documentados. Entretanto, são raros os estudos associando essa prática ao comportamento sedentário e ao risco cardiovascular em adolescentes.

**Objetivos:**

Pretende-se avaliar a prática de atividade física, o comportamento sedentário e a associação com o risco cardiovascular mensurado pelo escore *Pathobiological Determinants of Atherosclerosis in Youth* (PDAY).

**Métodos:**

Estudo transversal desenvolvido nas escolas públicas estaduais de Campina Grande, PB, Brasil, com 576 adolescentes de 15 a 19 anos, incluindo variáveis socioeconômicas, demográficas, de estilo de vida e clínicas. Os dados foram coletados através de formulário validado, antropometria, aferição da pressão arterial e exames laboratoriais. Foram utilizadas medidas descritivas, teste do qui-quadrado de Pearson e regressão logística binomial. Trabalhou-se com o SPSS 22.0 se adotou intervalo de confiança de 95%.

**Resultados:**

A idade média foi de 16,8 anos. A maioria dos adolescentes era do sexo feminino (66,8%), não branco (78.7%) e pertencente às classes C, D e (69,1%). Quanto ao sedentarismo e à insuficiência de atividade física, as prevalências foram de 78,1% e 60,2%, respectivamente. De acordo com o escore PDAY, 10,4% dos adolescentes apresentaram alto risco cardiovascular; 31,8% risco intermediário; e 57,8%, risco baixo. Verificou-se que PDAY esteve associado ao sexo e à adiposidade abdominal.

**Conclusões:**

Ficou comprovado que adiposidade abdominal e sexo masculino representam importantes fatores de risco cardiovascular em adolescentes. Considerando-se a presença de um fator de risco modificável, medidas preventivas voltadas ao estilo de vida são essenciais.

## INTRODUÇÃO

A atividade física (AF) corresponde a qualquer movimento realizado pelo corpo em que há dispêndio energético. Trata-se de um hábito importante para a manutenção da saúde, prevenção de doenças, bem-estar e desenvolvimento psicomotor, apresentando relação com o balanço energético e o controle da massa corporal^1^^,^ 2. A ausência de AF, denominada “inatividade”, corresponde ao quarto fator indireto de risco global para mortalidade. Sua prevalência tem aumentado em todo o mundo, bem como suas implicações no incremento das doenças crônicas não transmissíveis, como as doenças cardiovasculares (DCV)^3^^,^ 4.

Além disso, atualmente, há necessidade de avaliar a exposição tanto a baixo nível de AF quanto aos comportamentos sedentários (CS). Isso é importante porque existem evidências sugerindo que a inatividade física e o sedentarismo são comportamentos independentes e têm diferentes efeitos sobre a saúde^5^. O último está associado ao uso de equipamentos eletrônicos (computador, televisão e/ou videogame) por tempo igual ou superior a 2 horas diárias, definido como “tempo de tela”^6^.

Embora estejam associados a um aumento da morbimortalidade por DCV, ambos representam fatores de risco modificáveis, e que são mais bem-sucedidos na prevenção dessas morbidades quando implementados em fases precoces do ciclo vital. A DCV tem um longo período de latência, porém o surgimento dos fatores de risco (alterações no metabolismo lipídico, hipertensão arterial, resistência insulínica, tabagismo, inatividade física e obesidade) é precoce^7^.

Durante a adolescência, observa-se que a presença de dois ou mais fatores de risco é suficiente para a predição de um evento cardiovascular nos próximos 10 anos. Isso porque tais fatores, quando unidos, elevam a extensão e a gravidade das lesões vasculares, prevalecendo na fase adulta^8^. Com base nessa perspectiva, foram criados escores de estratificação de risco cardiovascular (RCV) capazes de predizer a ocorrência futura de eventos cardíacos patológicos.

O escore *Pathobiological Determinants of Atherosclerosis in Youth* (PDAY) estratifica precocemente (indivíduos de 15 a 34 anos) o risco para doença aterosclerótica e estabelece como premissa que fatores de risco para DCV estão associados, décadas antes do desfecho cardiovascular, com ambas as fases (inicial e avançada) das lesões ateroscleróticas na carótida e na aorta abdominal durante a adolescência e início da vida adulta^9^^-^ 11.

A estratificação do risco é obtida pela soma dos valores atribuídos a fatores modificáveis – colesterol não HDL, colesterol HDL, tabagismo, pressão arterial, índice de massa corporal (IMC), glicemia de jejum (GJ) e hemoglobina glicosilada HBA1c – e não modificáveis avaliados (idade, sexo). Se o resultado obtido for superior a zero, ele deve ser plotado no gráfico da probabilidade estimada para lesões ateroscleróticas graves na carótida e na aorta abdominal, órgãos-alvo^8^^,^ 10.

Tal escore é normalizado de modo que o aumento de uma unidade é equivalente a uma alteração exponencial positiva nas chances de lesão. Outro ponto relevante corresponde à idade. Para cada aumento de 5 anos na idade, o mesmo valor de pontos é adicionado. Assim, valores atribuídos aos fatores de risco modificáveis são equivalentes a 11 anos^8^^,^ 10.

Diante do exposto, e considerando a relativa escassez de estudos que utilizam os critérios do PDAY para a estratificação do RCV em adolescentes, e ainda sendo relevante verificar a sua aplicação e associação simultânea com o padrão de AF e com a exposição ao CS, este estudo visa identificar a prevalência destes em adolescentes e a associação dessas variáveis com o RCV mensurado pelo escore PDAY.

## MÉTODOS

Estudo transversal desenvolvido em escolas públicas estaduais de ensino médio da zona urbana municipal entre setembro de 2012 e junho de 2013. A população-alvo deste estudo foi constituída por 9.294 escolares com idade entre 15 e 19 anos matriculados em 264 turmas do ensino médio. O cálculo amostral considerou a estimativa de proporção de 50%, erro amostral de 5%, efeito do desenho (deff) de 1,5 (fator de correção para amostragem aleatória por conglomerado) e um acréscimo de 3% para eventuais perdas ou recusas, resultando na estimativa de 570 adolescentes.

A seleção de participantes ocorreu pela inexistência de situações, permanentes ou temporais, que prejudicassem a prática de AF ou comprometessem a realização dos procedimentos do estudo: gravidez; doença subjacente que cursasse com alteração do metabolismo dos lipídeos e/ou da glicemia, sendo incluídos 583 adolescentes. No entanto, sete desses recusaram-se a participar de alguma etapa do estudo, perfazendo, ao final, 576 adolescentes avaliados em 39 turmas de 18 escolas.

Para a coleta de dados, foi utilizado formulário para as variáveis socioeconômicas, demográficas e de estilo de vida. Para obtenção da massa corporal, foi utilizada balança digital Tanita® com capacidade para 150 kg e precisão de 0,1 kg. A estatura foi aferida através de estadiômetro portátil Tonelli® com precisão de 0,1 cm. A circunferência abdominal foi avaliada com fita métrica inelástica Cardiomed® com precisão de 0,1 cm no ponto médio entre a extremidade da última costela e a crista ilíaca, onde a fita métrica foi posicionada horizontalmente e mantida de tal forma que permanecesse na posição ao redor do abdômen sobre o nível da cicatriz umbilical, para que se procedesse à leitura da circunferência no milímetro mais próximo. Para aferição da pressão arterial foram utilizados aparelhos semiautomáticos OMRON–HEM 705CP®, seguindo as recomendações relatadas nas VI Diretrizes Brasileiras de Hipertensão Arterial^12^. A coleta sanguínea ocorreu sempre no período da manhã, após jejum prévio de 12 horas.

A idade foi avaliada em anos e o sexo como masculino/feminino. A cor foi categorizada em “branca” e “não branca”. A escolaridade materna foi verificada em anos completos e classificada em duas categorias: menor que 9 anos e 9 anos ou mais^13^. A classe econômica foi definida pelo escore construído pela soma da pontuação referente à posse e à quantidade de bens de consumo, empregada mensalista e grau de instrução do chefe de família e correspondente a uma determinada renda mensal familiar, definida pelos seguintes pisos: A1 = R$ 12.926,00; A2 = R$ 8.418,00; B1 = R$ 4.418,00; B2 = R$ 2.565,00; C1 = R$1.541,00; C2 = R$ 1.024,00; D = R$ 714,00; E = R$ 477,00^14^. A escolaridade materna foi verificada em anos completos e classificada em duas categorias: menor que 9 anos e 9 anos ou mais^13^.

O tabagismo foi classificado considerando-se duas categorias: fumante atual (pelo menos um cigarro/dia nos últimos 6 meses) e nunca fumou, uma vez que sua relação com alterações no metabolismo lipídico se dá apenas quando consumidas 11 unidades diárias^15^.

A prática de AF correspondeu à AF acumulada, combinando os tempos e as frequências com que foram realizadas atividades como: deslocamento para a escola (a pé ou de bicicleta), aulas de educação física na escola e outras atividades físicas extraescolares. Para a análise, os inativos e insuficientemente ativos I (até 149 minutos/semana) compuseram uma categoria, enquanto os insuficientemente ativos II (150 minutos ou mais/semana) e ativos (≥ 300 minutos/semana) constituíram a segunda. Foi considerado sedentário o adolescente que tivesse 2 ou mais horas/dia gastas no “tempo de tela”^6^.

O estado nutricional foi classificado de acordo com o IMC, construído a partir da razão do peso (em quilogramas) pelo quadrado da estatura (em metros). Foi utilizado para classificação do estado nutricional, de acordo com o escore *z* do IMC, segundo a idade e sexo: baixo peso (-3 ≤ escore *z* < -2), eutrofia (-2 ≤ escore *z* < +1), sobrepeso (+ 1 ≤ escore *z* < +2), obesidade (+2 ≤ escore *z* < +3) e obesidade acentuada (escore z ≥ +3). Para os maiores de 18 anos, os pontos de corte do IMC (em kg/m^2^) foram: baixo peso (< 17,5), eutrofia (17,5 ≤ IMC < 25,0), sobrepeso (25,0 ≤ IMC < 30,0) e obesidade (≥ 30,0)^16^^,^ 17. A circunferência abdominal foi classificada como aumentada para valores iguais ou superiores ao percentil 90^18^, porém com limite mínimo de 88 cm para mulheres e de 102 cm para os homens, de acordo com o *National Cholesterol Education Program Adult Treatment Panel* III^19^.

A pressão arterial elevada foi caracterizada pelos valores de pressão arterial sistólica e/ou diastólica iguais ou superiores ao percentil 95 para idade, sexo e percentil de estatura, de acordo com as tabelas específicas. Além disso, os valores de pressão arterial sistólica e diastólica iguais ou superiores a 120 mmHg e/ou 80 mmHg, respectivamente, foram considerados elevados, independente do percentil 95, para os adolescentes com 17 anos ou menos, após determinação prévia dos percentis de estatura pelos gráficos de desenvolvimento. A partir desta idade, considerou-se elevada a pressão arterial sistólica ≥ 130 mmHg e/ou diastólica ≥ 85 mmHg, independentemente do percentil^12^.

Foram avaliadas as variáveis bioquímicas necessárias à construção do escore de RCV pelo PDAY e, portanto, foram utilizados os critérios de referência desse escore: GJ ≥ 126 mg/dL, HDL-c < 40 mg/dL e colesterol não HDL > 130 mg/dL^20^. O ponto de corte da hemoglobina glicada HBA1c foi ajustado para referência mais atualizada, respeitando a pontuação do escore (> 6,5%)^21^.

A estratificação de risco foi construída pelo somatório de pontos, assim atribuídos: idade = 0 (adolescentes), sexo (masculino = 0, feminino = -1), não HDL-colesterol (< 130 mg/dL = 0; ≥ 130 mg/dL = 2 a 8), HDL-colesterol (< 40 mg/dL = 1; 40 a 59 mg/dL = 0; ≥ 60 mg/dL = -1), tabagismo (não = 0; sim = 1), pressão arterial (normal = 0; alterada = 1), IMC (pontua apenas para homens, quando > 30 kg/m^2^ = 6), e hiperglicemia (GJ < 126 mg/dL e HbA1c < 6,5% = 0; GJ ≥ 126 mg/dL e HbA1c ≥6, 5% = 5). Sobre o cálculo e análise de resultados, os adolescentes foram classificados como de baixo risco quando o somatório totalizou entre 0 e 1; de risco intermediário, entre 1 e 4; e de alto risco, se maior ou igual a 5^21^. Somatórios inferiores a zero foram considerados inadequados à estratificação do risco de lesão aterosclerótica^22^, uma vez que correspondem quantitativamente a perfis íntegros de sanidade e, por conseguinte, de baixo risco.

Os dados foram analisados no SPSS, 22.0. Foram utilizadas medidas descritivas, teste do qui-quadrado de Pearson, teste exato de Fisher (quando necessário) e regressão logística binomial para verificar a associação entre variáveis independentes e RCV. Adotou-se intervalo de confiança de 95%. O estudo foi aprovado pelo Comitê de Ética em Pesquisa da Universidade Estadual da Paraíba (CAEE: 0077.0.133.000-12).

## RESULTADOS

A amostra estudada foi de 576 escolares adolescentes de Campina Grande (PB), Brasil. Dentre os adolescentes avaliados, a média etária foi de 16,8 (±1,0) anos. A maioria era do sexo feminino (66,8%), não branco (78,7%), com menos de 9 anos de escolaridade materna (58,1%), e pertencentes às classes econômicas C, D e E (69,1%).

Dentre as mulheres, 79,5% (n = 299) consideraram-se não brancas, 56,6% tinham escolaridade materna superior a 9 anos, e 71.7% pertenciam às classes econômicas C, D e E. Da mesma forma, os homens identificaram-se, na maioria, como não brancos (77,1%) e pertenciam às classes C, D e E (63,9%). Nenhum adolescente foi classificado como pertencente à classe A1, e não foi observada diferença estatisticamente significante entre os sexos (Tabela 1).

**Tabela 1:** Distribuição dos adolescentes de acordo com as características socioeconômicas e demográficas, segundo o sexo. Campina Grande, PB, Brasil (2012-2013).

**Variável**	**Masculino**		**Feminino**		**RP**	**p**	**IC 95%**
	**n**	**%**	**n**	**%**			
Cor da pele (n = 563)^*^					0,968	0,512	0,881-1,063
Branca	43	23,0	77	20,5			
Não-branca	144	77,0	299	79,5			
Total	187	100	376	100			
Escolaridade materna (n = 568)^*^					0,832	0,312	0,583-1,188
≤ 9 anos	116	61,1	214	56,6			
> 9 anos	74	38,9	164	43,4			
Total	190	100	378	100			
Classe econômica					0,891	0,068	0,787-1,008
E a C1	122	63,9	276	71,7			
B2 a A2	69	36,1	109	28,3			
Total	191	100	385	100			

n = frequência absoluta; RP: razão de prevalência; *p*-valor: erro α de 5%; IC95%: intervalo de confiança de 95%;

* A variabilidade do n deve-se aos estudantes que não quiseram ou não souberam responder às questões.

### Variáveis relacionadas ao estilo de vida

Verificou-se que ser do sexo masculino favorece a prática de atividade física igual ou superior a 150 minutos semanais - razão de prevalência (RP): 0,484; intervalo de confiança de 95% (IC95%): 0,340-0,688. Quanto ao sedentarismo e ao tabagismo, como fatores de RCV^15^, não se verificaram diferenças entre os sexos (Tabela 2).

**Tabela 2:** Distribuição dos adolescentes quanto ao estilo de vida, segundo o sexo. Campina Grande, PB, Brasil (2012-2013).

**Variável**	**Masculino**		**Feminino**		**RP**	**p**	**IC95%**
	**n**	**%**	**n**	**%**			
Prática de atividade física					0,484	< 0,01	0,340-0,688
0 a 149 minutos/semana	31	16,2	129	33,5			
≥ 150 minutos/semana	160	83,8	256	66,5			
Total	191	100	385	100			
Sedentarismo					0,945	0,239	0,859-1,040
Não	47	24,6	78	20,3			
Sim	144	75,4	307	79,7			
Total	191	100	385	100			
Tabagismo (n = 575)*					1,675	0,584†	0,518-5,420
Não	186	97,4	378	98,4			
Sim	5	2,6	6	1,6			
Total	191	100	384	100			

n = frequência absoluta; RP: razão de prevalência; *p*-valor: erro α de 5%; IC95%: intervalo de confiança de 95%;

* A variabilidade do n deve-se a um estudante que não quis ou não soube responder à questão;

† Teste exato de Fisher.

### Variáveis clínicas e bioquímicas

A maioria das mulheres apresentou não HDL-colesterol (81,3%) e HDL colesterol (66,8%) dentro dos valores desejáveis. Ser do sexo masculino aumenta em quase duas vezes o risco de ter HDL-c alterado (RP: 1,748; IC95%: 1,452-2,105) e em três vezes a pressão arterial aumentada (RP: 3,001; IC95%: 2,145-4,198).

A classificação do estado nutricional registrou apenas dois adolescentes com baixo peso (0,4%), sendo a maioria eutrófica (62,8%), 44,1% com sobrepeso e 7,3% obesos (dados não tabulados). Para fins de análise, o estado nutricional foi reagrupado: baixo peso e eutrofia passaram a formar uma categoria (63,2%), e sobrepeso e obesidade, outra (36,8%). Quanto à circunferência abdominal, 3,3% da amostra apresentaram alteração. O perfil glicídico, para a maioria dos adolescentes, estava dentro da normalidade (Tabela 3).

**Tabela 3:** Distribuição dos adolescentes quanto aos fatores de risco cardiovasculares constituintes do escore PDAY, segundo o sexo. Campina Grande, PB, Brasil (2012-2013).

**Variáveis**	**Masculino**		**Feminino**		**RP**	**p**	**IC95%**
	**n**	**%**	**n**	**%**			
Colesterol não-HDL (mg/dL)					0,699	0,098	0,459-1,065
Alterado ≥ 130	25	13,1	72	18,7			
Desejável < 130	166	86,9	313	81,3			
Total	191	100	385	100			
Colesterol HDL (mg/dL)					1,748	< 0,01	1,452-2,105
Alterado < 40	111	58,1	128	33,6			
Desejável ≥ 40	80	41,9	257	66,8			
Total	191	100	385	100			
Pressão arterial (mmHg)					3,001	< 0,01	2,145-4,198
Alterado	67	35.1	45	11.7			
Normal	124	64.9	340	88.3			
Total	191	100	385	100			
Estado nutricional (escore z)					1,028	0,906	0,652-1,620
Sobrepeso/obeso	34	17,8	67	17,4			
Baixo peso/eutrófico	157	82,2	318	82,6			
Total	191	100	385	100			
Circunferência abdominal (cm)					0,928	0,882	0,347-2,481
Alterado	6	3,1	13	3,4			
Normal	185	96,9	372	96,6			
Total	191	100	385	100			
Glicemia de jejum (mg/dL)					-	-	-
Alterada	-	-	1	-			
Normal	191	100	385	100			
Total	191	100	386	100			
Hemoglobina glicosilada (%)					-	0,718*	-
Alterado	1	0,5	1	0			
Normal	189	99,5	385	100			
Total	190	100	386	100			

n = frequência absoluta; RP: razão de prevalência; *p*-valor: erro α de 5%; IC95%: intervalo de confiança de 95%;

* Teste exato de Fisher.

### Estilo de vida *versus* risco cardiovascular

De acordo com o escore global de RCV (PDAY), registraram-se baixo RCV para 57,8% da amostra, risco intermediário para 31,8%, e alto risco para 10,4%. Para fins de análise, o escore foi reagrupado: alto risco e risco intermediário (42,2%) formaram uma categoria, e baixo risco (57,8%), a outra. Os adolescentes do sexo masculino foram maioria quando considerados os escores alto e intermediário (76,7%), já o escore PDAY baixo prevaleceu entre as mulheres (71,9%). Verificou-se que o sexo e a circunferência abdominal representam fatores de risco para o escore PDAY intermediário e alto, ao passo que a prática de AF igual ou superior a 150 minutos semanais representou um fator de proteção (Tabela 4).

**Tabela 4:** Análise bivariada dos fatores socioeconômicos, demográficos, de estilo de vida e clínicos, segundo o escore de risco PDAY. Campina Grande, PB, Brasil (2012-2013).

**Variáveis**	**Risco cardiovascular intermediário e alto** **(n = 60)**		**Risco cardiovascular baixo** **(n = 516)**		**RP**	**p**	**IC95%**
	**n**	**%**	**n**	**%**			
Sexo					8,407	< 0,01	4,485-15,758
Masculino	46	76,7	145	28,1			
Feminino	14	23,3	371	71,9			
Total	60	100	516	100			
Cor da pele (n = 563)*					1,337	0,424	0,655-2,728
Não-branca	48	82.8	395	78.2			
Branca	10	17.2	110	21.8			
Total	58	100	505	100			
Escolaridade materna (n = 568)*					0,783	0,385	0,450-1,362
≤ 9 anos	38	63,3	292	57,5			
> 9 anos	22	36,7	216	42,5			
Classe econômica					1,145	1,000	0,394-3,322
E a C1	56	93,3	477	92,4			
B2 a A2	4	6,7	39	7,6			
Total	60	100	516	100			
Prática de atividade física					0,448	0,042	0,241-0,988
Inativo ou insuficientemente ativo I (0 a 149 min/semana)	10	16,7	150	29,1			
Insuficientemente ativo II e ativo (≥ 150 min/semana)	50	83,3	366	70,9			
Total	60	100	516	100			
Sedentarismo (horas/dia)					1,122	0,736	0,576-2,183
≥ 2 horas/dia	48	80,0	403	78,1			
< 2 horas/dia	12	20,0	113	21,9			
Total	60	100	516	100			
Circunferência abdominal (cm)					11,267	< 0,01	4,374-29,023
Alterada	10	16,7	9	1,7			
Normal	50	83,3	507	98,3			
Total	60	100	516	100			

n = frequência absoluta; RP: razão de prevalência; *p*-valor: erro α de '5%; IC95%: intervalo de confiança de 95%;

* A variabilidade do n deve-se aos estudantes que não quiseram ou não souberam responder à questão.

Quando avaliada conjuntamente, a relação da AF com o RCV deixou de ser significante. Dessa forma, permaneceram no modelo final o sexo e a circunferência abdominal. Ser do sexo feminino e ter circunferência abdominal adequada implicam em uma menor probabilidade de apresentar RCV intermediário ou alto (Tabela 5).

**Tabela 5:** Análise de regressão logística do risco cardiovascular mensurado pelo escore PDAY e variáveis preditoras. Campina Grande, PB, Brasil (2012-2013).

**Variável de saída**	**Variáveis preditoras**	**R^2^**	**B(Coef)**	**p**	**IC95%**	HL*
Risco cardiovascular (PDAY)	Sexo	0,271	0,090	< 0,01	0,044-0,183	0.939
	Circunferência abdominal		0,043	< 0,01	0,014-0,136	

R^2^ de Nagelkerke: ajuste; B(Coef): coeficiente beta; *p*-valor: erro α de 5%; IC95%: intervalo de confiança de 95%; *Teste de Hosmer e Lemeshow.

## DISCUSSÃO

Este estudo retrata o padrão de AF, bem como o padrão de exposição ao CS, identificando suas prevalências nos escolares e estabelecendo a relação desses padrões com o RCV mensurado pelo escore PDAY. Foi observado que o tempo dispendido na prática de AF foi predominantemente (77,2%) superior a 150 minutos/semanais, sendo essa prática mais prevalente entre os homens (83,8%) e alterando-se segundo o sexo. Estudos recentes também atestaram que essa prática se apresenta menos prevalente no sexo feminino entre os adolescentes^23^^-^ 27, seguindo a tendência nacional^6^ e mundial^4^^,^ 28 ^,^ 29.

A influência do sexo sobre a prática de AF tem sido objeto de diversos estudos^4^^,^ 23 ^,^ 24 ^,^ 27 que afirmam que o sexo feminino é menos ativo se considerados: os menores níveis de escolaridade dos genitores, refletindo a ausência de apoio e incentivo a essa prática; o menor patamar socioeconômico, resultando em menor acesso a atividades de maior gasto energético; além da preferência feminina por atividades individuais e leves^26^. Contudo, estudo brasileiro que analisou a prevalência de insuficiente prática de AF entre adolescentes não observou diferença entre os sexos^30^.

Ao verificar a relação da AF com o RCV, constatou-se que aquela mostra associação quando avaliada isoladamente, embora perca a significância no modelo de regressão ao ser tratada junto com o sexo e a circunferência abdominal. Outros estudos que analisaram tal relação^4^^,^ 29 também afirmam que os benefícios atrelados à AF promovem proteção contra fatores de risco cardiometabólicos (dislipidemia, resistência insulínica, hipertensão).

Quanto ao CS, sua prevalência na amostra foi de 78.3%, predominando entre as mulheres (79,7%). Ao contrário dos nossos achados, estudo pernambucano^23^ mostrou que, embora mais sedentários, os homens também foram mais ativos, de forma que o CS não influenciou o nível de AF masculina. Por outro lado, uma amostra estudada também em Pernambuco evidenciou que o CS influencia negativamente a prática de AF, em especial a feminina^26^.

Ao explorar a relação entre CS e RCV, constatou-se a inexistência de relação estatística entre as variáveis. Tal achado se contrapõe ao do estudo AFINOS, que calculou separadamente o CS^9^, no qual o elevado tempo de TV acarretou a presença de moléculas de adesão, de marcadores de processos ateroscleróticos, e de instabilidade de placas ateroscleróticas. Esses achados foram reiterados em estudo de revisão^10^. Sendo assim, é provável que a ausência de relação observada se deva à questão de o tempo de tela ter sido avaliado na íntegra.

Ademais, quanto aos fatores de RCV segundo o sexo, identificou-se que os níveis séricos de colesterol HDL e a pressão arterial estavam inadequados predominantemente nos homens; enquanto que o sexo, a prática de AF e a adiposidade abdominal foram associados ao RCV. Em estudo desenvolvido por Ribas & Silva, o sexo masculino (mais ativo) apresentou menor probabilidade de desenvolver hipertensão arterial sistêmica, ao passo que o feminino (mais inativo e sedentário) não apresentou risco de dislipidemia. Isso se deve aos hormônios sexuais femininos atuarem como fator de proteção ao RCV^8^.

Na análise de regressão, apenas o sexo e a adiposidade abdominal permaneceram como fatores que implicam uma menor probabilidade de apresentar RCV intermediário ou alto. Esse fato ratifica a descoberta de Shah et al.^11^, que também trabalharam com o escore PDAY, atestando que o sexo feminino teve menor RCV e esteve associado a uma menor adiposidade abdominal. Além disso, esta representa fator preditivo independente de RCV^31^, sendo objeto de estudo de revisão que confirmou a atuação da gordura ectópica na liberação de adipocitinas, lipotoxinas e glicotoxinas que acarretam disfunções cardiovasculares^8^.

O presente estudo apresenta a importante característica de ser de base populacional, utilizando instrumentos confiáveis, e ser o pioneiro na análise do RCV mensurado pelo escore PDAY em adolescentes brasileiros. Como limitação, por se tratar de um estudo transversal, não permite estabelecer relação causal entre as variáveis estudadas e o escore de risco PDAY – deixando essa pergunta para que novos estudos possam ser desenvolvidos, visando a prevenção precoce das DCVs.
