# Supplementary material for: Adolescentes: comportamento e risco cardiovascular
Source: J Vasc Bras. 2017 Jul-Sep;16(3):205–13. [Article in Portuguese] doi: 10.1590/1677-5449.011816 (PMC5868936; doi:10.1590/1677-5449.011816)
Supplement: Translation

# Translation: Adolescents: behavior and cardiovascular risk

**Authors:** Ivelise Fhrideraid Alves Furtado da Costa, Carla Campos Muniz Medeiros, Fernanda Dayenne Alves Furtado da Costa, Camilla Ribeiro Lima de Farias, Diogo Rodrigues Souza, Wellington Sabino Adriano, Mônica Oliveira da Silva Simões, Danielle Franklin Carvalho

**Affiliations:** 1 Universidade de Pernambuco – UPE, Faculdade de Enfermagem Nossa Senhora das Graças, Recife, PE, Brazil.; 2 Universidade Estadual da Paraíba – UEPB, Programa de Mestrado em Saúde Pública, Campina Grande, PB, Brazil.; 3 Universidade Federal de Campina Grande – UFCG, Centro de Educação e Saúde, Campina Grande, PB, Brazil.; 4 Centro Universitário Unifacisa, Departamento de Enfermagem, Campina Grande, PB, Brazil.

**Keywords:** physical activity, sedentary behavior, cardiovascular diseases, adolescents

## Abstract

**Background:**

The health benefits of regular physical activity are well documented. However, there are few studies associating this practice with sedentary behavior and cardiovascular risk in adolescents.

**Objectives:**

To evaluate physical activity levels and sedentary behavior and their associations with cardiovascular risk using the Pathobiological Determinants of Atherosclerosis in Youth (PDAY) score

**Methods:**

A cross-sectional study carried out in state-owned public schools in Campina Grande, PB, Brazil, with 576 adolescents aged 15 to 19 years, investigating socioeconomic; demographic; lifestyle; and clinical variables. Data were collected using a validated form covering anthropometry data; blood pressure measurements; and laboratory tests. Data were analyzed using descriptive statistics, Pearson's chi-square test, and binomial logistic regression using SPSS 22.0 and adopting a 95% confidence interval.

**Results:**

Mean age was 16.8 years. The majority of the adolescents were female (66.8%); non-white (78.7%); and belonged to socioeconomic classes C, D and E (69.1%). The prevalence rates of sedentary behavior and physical inactivity were 78.1% and 60.2%, respectively. According to the PDAY score, 10.4% of adolescents were at high cardiovascular risk and 31.8% and 57.8% were at intermediate risk and low risk, respectively. PDAY scores were associated with sex and abdominal adiposity.

**Conclusions:**

It was found that abdominal fat and being male were important cardiovascular risk factors in adolescents. Considering that modifiable risk factors were present, preventive measures aimed at lifestyle changes are essential.

## INTRODUCTION

Physical activity (PA) is any movement of the body that involves energy expenditure. It is an important habit for maintenance of health, prevention of diseases, and promotion of wellbeing and psychomotor development, and it has a relationship with energy balance and control of body mass.^1^^,^ 2 Absence of PA, known as “inactivity”, has been identified as the fourth ranked indirect risk factor for mortality worldwide. The prevalence of inactivity has increased all over the world and so have its consequences in terms of increased rates of non-transmissible chronic diseases, such as cardiovascular diseases (CVD).^3^^,^ 4

Furthermore, it is now necessary to investigate exposure both to low PA levels and to sedentary behaviors (SB). This is important because there is evidence that physical inactivity and sedentary habits are independent behaviors and have different effects on health.^5^ The second of these is related to use of electronic equipment (computers, televisions, and/or video games) for 2 hours or more per day, defined as “screen time”.^6^

While both of these phenomena are associated with increased morbidity and mortality due to CVD, they are modifiable risk factors and interventions to alter them are more successful at preventing these morbidities when they are implemented early on during the life cycle. Cardiovascular diseases have a long latency period, but the risk factors (lipid metabolism abnormalities, arterial hypertension, insulin resistance, smoking, physical inactivity, and obesity) have early onset.^7^

It has been observed that presence of two or more risk factors during adolescence is sufficient to predict a cardiovascular event during the following 10 years. This is because when these factors are present in combination they increase the extent and severity of vascular injuries, which predominantly emerge during adulthood.^8^ In response to these findings, scores for stratification of cardiovascular risk (CVR) have been developed and shown to be capable of predicting future occurrence of pathological cardiac events.

The Pathobiological Determinants of Atherosclerosis in Youth (PDAY) score was developed for early (among people aged 15 to 34 years) stratification of risk of atherosclerotic disease and is based on the premise that decades before any cardiovascular outcomes emerge, risk factors for CVD are already associated with both phases (initial and advanced) of atherosclerotic lesions involving the carotids and abdominal aorta during adolescence and early adulthood.^9^^-^ 11

Risk stratification is achieved by summing values attributed for modifiable factors – non-HDL cholesterol, HDL cholesterol, smoking, arterial blood pressure, body mass index (BMI), fasting glycemia (FG), and glycosylated hemoglobin HBA1c – and values attributed for non-modifiable factors (age, sex). If the result is greater than zero, it should be plotted on the graph of estimated probability of severe atherosclerotic lesions of the carotids and abdominal aorta, the target-organs.^8^^,^ 10

The score is normalized so that a unit increase is equivalent to a positive exponential change in the likelihood of injury. Another relevant point is related to age. For each 5-year increment in age, the same value is added in points. Therefore, the values attributed to modifiable risk factors are the equivalent of 11 years.^8^^,^ 10

In view of all of the above, and considering the relative scarcity of studies that have employed the PDAY criteria to stratify CVR in adolescents, in addition to the relevance of testing its application and simultaneous association with PA patterns and with exposure to SB, the objectives of this study are to identify the prevalence of these factors in adolescents and to test for associations between these variables and CVR measured using the PDAY score.

## METHODS

A cross-sectional study was conducted at state-run public secondary schools in an urban municipal zone from September 2012 to June 2013. The target population of the study comprised 9,294 schoolchildren aged 15 to 19 years and enrolled in 264 classes at secondary schools. The sample size was calculated based on an estimated proportion of 50%, a 5% sampling error, and a design effect of 1.5 (correction factor for randomized cluster sampling), and then increased by 3% to account for possible losses or refusals, giving an estimate of 570 adolescents.

Participants were selected if they did not have any permanent or temporary conditions that would interfere with PA or compromise the study procedures (pregnancy or underlying diseases that involve abnormalities of lipid metabolism and/or glycemia), and 583 adolescents were selected. However, seven of them refused to participate in at least one of the stages of the study, so the final sample comprised 576 adolescents assessed, enrolled in 39 classes from 18 schools.

Data on socioeconomic and demographic variables and lifestyle were collected using a form. Body mass was measured using a Tanita® digital balance with 150 kg capacity and precision of 0.1 kg. Height was measured using a Tonelli® portable stadiometer with precision of 0.1 cm. Waist circumference was measured with a Cardiomed® inextensible tape measure with precision of 0.1 cm, at the midpoint between the extremity of the last rib and the iliac crest, where the tape measure was positioned horizontally and placed to run around the abdomen at the level of the umbilical scar, and the circumference was read off to the closest millimeter. Arterial blood pressure was measured using OMRON–HEM 705CP® semiautomatic meters, following the recommendations described in the VI Brazilian Guidelines on Arterial Hypertension.^12^ All blood samples were drawn in the morning after a fasting period of 12 hours.

Age was analyzed in years and sex recorded as male/female. Skin color was categorized as “white” or “not white”. Mother’s educational level was recorded in full years’ schooling and classified into two categories: less than 9 years and 9 years or more.^13^ Economic class was defined according to a score composed of the sum of points for possession and number of consumer goods, whether the family has maids at home, and educational level of the head of the family, corresponding to a given monthly family income, defined by the following lower limits: A1 = R$ 12,926.00; A2 = R$ 8,418.00; B1 = R$ 4,418.00; B2 = R$ 2,565.00; C1 = R$1,541.00; C2 = R$ 1,024.00; D = R$ 714.00; and E = R$ 477.00.^14^ Mother’s educational level was recorded in full years’ schooling and classified into two categories: less than 9 years and 9 years or more.^13^

Smoking status was classified into two categories: current smoker (at least one cigarette/day over the previous 6 months) and never smoked, since its relationship to lipid metabolism abnormalities only occurs when 11 units per day are consumed.^15^

Physical activity level was defined as all PA accumulated by combining durations and frequencies of activities such as: displacement to school (on foot or by bicycle), Physical Education classes at school, and other extramural physical activities. For the purposes of analysis, adolescents classed as inactive or as insufficiently active I (up to 149 minutes/week) were collapsed to form one category, and insufficiently active II (150 minutes or more/week) and active (≥ 300 minutes/week) adolescents were grouped together in a second category. Adolescents who reported 2 hours or more of “screen time” per day were defined as sedentary.^6^

Nutritional status was classified according to BMI, calculated by taking the ratio of weight (in kilograms) to the square of height (in meters). The result was used to classify nutritional status according to BMI z scores for age and sex: underweight (-3 ≤ z score < -2), healthy weight (-2 ≤ z score < +1), overweight (+ 1 ≤ z score < +2), obesity (+2 ≤ z score < +3), and accentuated obesity (z score ≥ +3). For participants over the age of 18 years, BMI cutoff points (kg/m^2^) were: underweight (< 17.5), healthy weight (17.5 ≤ BMI < 25.0), overweight (25.0 ≤ BMI < 30.0), and obesity (≥ 30.0).^16^^,^ 17 Waist circumference values greater than or equal to the 90th percentile were classified as elevated,^18^ but with minimum cutoffs of 88 cm for women and 102 cm for men, as per the National Cholesterol Education Program Adult Treatment Panel III.^19^

High blood pressure was defined as systolic and/or diastolic blood pressure values greater than or equal to the 95th percentiles for age, sex, and height percentile, according to relevant tables. Additionally, systolic and diastolic blood pressures greater than or equal to 120 mmHg and/or 80 mmHg respectively were defined as elevated, irrespective of the 95th percentile, for adolescents aged 17 or less, after determination of percentiles for height according to growth curves. Beyond 17 years of age, systolic blood pressures ≥ 130 mmHg and/or diastolic pressures ≥ 85 mmHg were defined as elevated irrespective of percentile.^12^

The biochemical variables that comprise the CVR score according to the PDAY were assessed and the reference criteria from the score were used, as follows: FG ≥ 126 mg/dL, HDL-c < 40 mg/dL, and non-HDL cholesterol > 130 mg/dL.^20^ The cutoff point for HBA1c glycated hemoglobin was altered to a more recent reference level, maintaining the number of points allocated by the score (> 6.5%).^21^

Risk stratification was conducted according to the sum of points allocated as follows: age = 0 (adolescents), sex (male = 0, female = -1), non-HDL cholesterol (< 130 mg/dL = 0; ≥ 130 mg/dL = 2 to 8), HDL-cholesterol (< 40 mg/dL = 1; 40 to 59 mg/dL = 0; ≥ 60 mg/dL = -1), smoking (no = 0; yes = 1), arterial blood pressure (normal = 0; abnormal = 1), BMI (scores for men only, when > 30 kg/m^2^ = 6), and hyperglycemia (FG < 126 mg/dL and HbA1c < 6.5% = 0; FG ≥ 126 mg/dL and HbA1c ≥ 6.5% = 5). Once the scores had been calculated, the results were used to classify adolescents as follows: low risk for total points from 0 to 1; intermediate risk, for 1 to 4 points; and high risk, if the total number of points was greater than or equal to 5 points.^21^ Total scores below zero were defined as inappropriate for stratification of risk of atherosclerotic lesions,^22^ since they correspond quantitatively to entirely healthy profiles and, therefore, to low risk.

Data were analyzed with SPSS 22.0, calculating descriptive statistics and performing Pearson’s chi-square test, Fisher’s exact test (when necessary), and binomial logistic regression to quantify associations between independent variables and CVR. A 95% confidence interval was adopted. The study was approved by the Research Ethics Committee at the Universidade Estadual da Paraíba (approval certificate number: 0077.0.133.000-12).

## RESULTS

The sample studied comprised 576 adolescent schoolchildren from Campina Grande, PB, Brazil. The mean age of the adolescents was 16.8 (±1.0) years. The majority were female (66.8%), non-white (78.7%), had mothers who had spent fewer than 9 years in education (58.1%), and were members of economic classes C, D, and E (69.1%).

Among female participants, 79.5% (n = 299) self-reported as non-white, 56.6% had mothers with educational level greater than 9 years, and 71.7% were members of economic classes C, D, and E. Similarly, the majority of the males self-reported as non-white (77.1%) and were from classes C, D and, E (63.9%). None of the adolescents were classified as class A1, and no statistically significant difference between the sexes was observed (Table 1).

**Table 1:** Distribution of the adolescents by socioeconomic and demographic characteristics, broken down by sex. Campina Grande, PB, Brazil (2012-2013).

**Variable**	**Male**		**Female**		**PR**	**p**	**95%CI**
	**n**	**%**	**n**	**%**			
Skin color (n = 563)*					0.968	0.512	0.881-1.063
White	43	23.0	77	20.5			
Non-white	144	77.0	299	79.5			
Total	187	100	376	100			
Mother’s educational level (n = 568)*					0.832	0.312	0.583-1.188
≤ 9 years	116	61.1	214	56.6			
> 9 years	74	38.9	164	43.4			
Total	190	100	378	100			
Economic class					0.891	0.068	0.787-1.008
E to C1	122	63.9	276	71.7			
B2 to A2	69	36.1	109	28.3			
Total	191	100	385	100			

n = absolute frequency; PR: prevalence ratio; *p*-value: α error of 5%; 95%CI: 95% confidence interval;

* Variability in n is the result of adolescents who did not wish to answer or were unable to answer certain questions.

### Variables related to lifestyle

It was observed that male sex was associated with weekly physical activity level greater than or equal to 150 minutes (prevalence ratio [PR]: 0.484; 95% confidence interval [95%CI]: 0.340-0.688). There were no differences between sexes in terms of inactivity or smoking as CVR factors^15^ (Table 2).

**Table 2:** Distribution of adolescents by lifestyle, broken down by sex. Campina Grande, PB, Brazil (2012-2013).

**Variable**	**Male**		**Female**		**PR**	**p**	**95%CI**
	**n**	**%**	**n**	**%**			
Physical activity level					0.484	< 0.01	0.340-0.688
0 to 149 minutes/week	31	16.2	129	33.5			
≥ 150 minutes/week	160	83.8	256	66.5			
Total	191	100	385	100			
Sedentary					0.945	0.239	0.859-1.040
No	47	24.6	78	20.3			
Yes	144	75.4	307	79.7			
Total	191	100	385	100			
Smoking (n = 575)*					1.675	0.584†	0.518-5.420
No	186	97.4	378	98.4			
Yes	5	2.6	6	1.6			
Total	191	100	384	100			

n = absolute frequency; PR: prevalence ratio; *p*-value: α error of 5%; 95%CI: 95% confidence interval;

* Variability in n is the result of adolescents who did not wish to answer or were unable to answer the question;

† Fisher’s exact test.

### Clinical and biochemical variables

The majority of female participants had non-HDL cholesterol (81.3%) and HDL cholesterol (66.8%) within the desirable range. Being male doubled the risk of abnormal HDL-c (PR:1.748; 95%, CI: 1.452-2.105) and increased the risk of high blood pressure threefold (PR: 3.001; 95%, CI: 2.145-4.198).

Nutritional status assessment classified two adolescents as underweight (0.4%), the majority as healthy weight (62.8%), 44.1% as overweight, and 7.3% as obese (data not shown in tables). For the purposes of analysis, nutritional status categories were regrouped: underweight and healthy weight were collapsed into a single category (63.2%), and overweight and obesity into another (36.8%). The waist circumferences of 3.3% of the sample were excessive. For most of the adolescents, glucose levels were within normal limits (Table 3).

**Table 3:** Distribution of adolescents by cardiovascular risk factors that comprise the PDAY score, broken down by sex. Campina Grande, PB, Brazil (2012-2013).

**Variables**	**Male**		**Female**		**PR**	**p**	**95%CI**
	**n**	**%**	**n**	**%**			
Non-HDL Cholesterol (mg/dL)					0.699	0.098	0.459-1.065
Abnormal ≥ 130	25	13.1	72	18.7			
Healthy < 130	166	86.9	313	81.3			
Total	191	100	385	100			
HDL cholesterol (mg/dL)					1.748	< 0.01	1.452-2.105
Abnormal < 40	111	58.1	128	33.6			
Healthy ≥ 40	80	41.9	257	66.8			
Total	191	100	385	100			
Arterial blood pressure (mmHg)					3.001	< 0.01	2.145-4.198
Abnormal	67	35,1	45	11,7			
Normal	124	64,9	340	88,3			
Total	191	100	385	100			
Nutritional status (z score)					1.028	0.906	0.652-1.620
Overweight/obese	34	17.8	67	17.4			
Underweight/healthy weight	157	82.2	318	82.6			
Total	191	100	385	100			
Waist circumference (cm)					0.928	0.882	0.347-2.481
Abnormal	6	3.1	13	3.4			
Normal	185	96.9	372	96.6			
Total	191	100	385	100			
Fasting glycemia (mg/dL)					-	-	-
Abnormal	-	-	1	-			
Normal	191	100	385	100			
Total	191	100	386	100			
Glycosylated hemoglobin (%)					-	0.718*	-
Abnormal	1	0.5	1	0			
Normal	189	99.5	385	100			
Total	190	100	386	100			

n = absolute frequency; PR: prevalence ratio; *p*-value: α error of 5%; 95%CI: 95% confidence interval;

* Fisher’s exact test.

### Lifestyle vs. cardiovascular risk

The total CVR (PDAY) scores indicated low CVR for 57.8% of the sample, intermediate risk for 31.8%, and high risk for 10.4%. For the purposes of analysis, these scores were regrouped as follows: high risk and intermediate risk (42.2%) were collapsed to a single category, and low risk (57.8%) was assigned to a second category. Male adolescents were in the majority in the high and intermediate scores group (76.7%), while females predominated in the low PDAY score group (71.9%). It was observed that sex and waist circumference were risk factors for a high or intermediate PDAY score, whereas weekly PA levels greater than or equal to 150 minutes was a protective factor (Table 4).

**Table 4:** Bivariate analysis of socioeconomic and demographic factors and lifestyle and clinical variables, by PDAY risk score. Campina Grande, PB, Brazil (2012-2013).

**Variables**	**Intermediate and high cardiovascular risk** **(n = 60)**		**Low cardiovascular risk** **(n = 516)**		**PR**	**p**	**95%CI**
	**n**	**%**	**n**	**%**			
Sex					8.407	< 0.01	4.485-15.758
Male	46	76.7	145	28.1			
Female	14	23.3	371	71.9			
Total	60	100	516	100			
Skin color (n = 563)*					1.337	0.424	0.655-2.728
Non-white	48	82.8	395	78.2			
White	10	17.2	110	21.8			
Total	58	100	505	100			
Mother’s educational level (n = 568)*					0.783	0.385	0.450-1.362
≤ 9 years	38	63.3	292	57.5			
> 9 years	22	36.7	216	42.5			
Economic class					1.145	1.000	0.394-3.322
E to C1	56	93.3	477	92.4			
B2 to A2	4	6.7	39	7.6			
Total	60	100	516	100			
Physical activity level					0.448	0.042	0.241-0.988
Inactive or insufficiently active I (0 to 149 min/week)	10	16.7	150	29.1			
Insufficiently active II or active (≥ 150 min/week)	50	83.3	366	70.9			
Total	60	100	516	100			
Sedentary behavior (hours/day)					1.122	0.736	0.576-2.183
≥ 2 hours/day	48	80.0	403	78.1			
< 2 hours/day	12	20.0	113	21.9			
Total	60	100	516	100			
Waist circumference (cm)					11.267	< 0.01	4.374-29.023
Abnormal	10	16.7	9	1.7			
Normal	50	83.3	507	98.3			
Total	60	100	516	100			

n = absolute frequency; PR: prevalence ratio; *p*-value: α error of 5%; 95%CI: 95% confidence interval;

* Variability in n is the result of adolescents who did not wish to answer or were unable to answer the question.

When variables were analyzed in conjunction, the relationship between PA and CVR was no longer significant. As such, sex and waist circumference were retained in the final model. Being female and having a healthy waist circumference were associated with a lower probability of intermediate or high CVR (Table 5).

**Table 5:** Logistic regression analysis of cardiovascular risk measured by the PDAY score and predictive variables. Campina Grande, PB, Brazil (2012-2013).

**Output variable**	**Predictive variables**	**R^2^**	**B(Coef)**	**p**	**95%CI**	**HL** ^*^
Cardiovascular risk (PDAY)	Sex	0.271	0.090	< 0.01	0.044-0.183	0,939
	Waist circumference		0.043	< 0.01	0.014-0.136	

Nagelkerke’s R^2^: fit. B(Coef): beta coefficient; *p*-value: α error of 5%; 95%CI: 95% confidence interval;

* Hosmer and Lemeshow Test.

## DISCUSSION

This study reports the profile of PA and exposure to SB, identifying their prevalence rates among schoolchildren and establishing the relationship between these profiles and CVR as measured by the PDAY score. It was observed that time spent engaged in PA was predominantly (77.2%) greater than 150 minutes/week, and that this was more prevalent among males (83.8%) and was different between the sexes. Other recent studies have also shown that PA is less prevalent among female adolescents than males,^23^^-^ 27 which is a tendency both nationally in Brazil^6^ and globally.^4^^,^ 28 ^,^ 29

The influence of sex on PA levels has been the subject of several studies^4^^,^ 23 ^,^ 24 ^,^ 27 that have reported that females are less active if the following are considered: lower educational level of parents, reflecting a lack of support and encouragement for the practice; lower socioeconomic levels, resulting in restricted access to activities with greater energy expenditure; and a preference among females for individual and lighter activities.^26^ Notwithstanding, one Brazilian study that analyzed the prevalence of insufficient PA among adolescents did not observe a difference between the sexes.^30^

Analysis of the relationship between PA and CVR revealed that there was an association when analyzed in isolation, but this association was not significant in the regression model when analyzed together with sex and waist circumference. Other studies that have analyzed this relationship^4^^,^ 29 have also reported that benefits linked to PA provide protection against cardiometabolic risk factors (dyslipidemia, insulin resistance, and hypertension).

The prevalence of SB in the sample was 78.3%, predominantly among the female adolescents (79.7%). In contrast to our findings, a study conducted in the Brazilian state of Pernambuco^23^ showed that men were both more sedentary and more active, so that SB did not affect the PA level of males. On the other hand, another sample, also studied in Pernambuco, provided evidence that SB had a negative impact on PA, especially among females.^26^

Analysis of the relationship between SB and CVR showed that there was no statistically significant association between these variables. This contradicts the AFINOS study, which calculated SB separately^9^ and found that high levels of TV time were linked with presence of adhesion molecules, markers of atherosclerotic processes, and instability of atherosclerotic plaques. Those findings were confirmed in a review study.^10^ It is therefore probable that the absence of a relationship observed in the present study is the result of screen time having been assessed in its entirety.

When CVR factors were analyzed by sex, it was found that serum HDL cholesterol levels and arterial blood pressure were predominantly at unhealthy levels among male adolescents; while sex, PA levels, and abdominal adiposity were associated with CVR. In a study conducted by Ribas and Silva, males (who were more active) had a lower probability of developing systemic arterial hypertension, whereas females (more inactive and more sedentary) did not have dyslipidemia risk. This is because female sex hormones acted as a factor of protection against CVR.^8^

In the regression analysis, only sex and abdominal adiposity were retained as factors that explained a lower probability of intermediate or high CVR. This fact confirmed a discovery made by Shah et al.,^11^ who also used the PDAY score and showed that females had lower CVR and that female sex was associated with lower abdominal adiposity. Additionally, abdominal adiposity is an independent predictive factor of CVR^31^ and was the subject of a review study that confirmed that ectopic fat is active in release of adipocytokines, lipotoxins, and glycotoxins that cause cardiovascular dysfunctions.^8^

This study has certain important characteristics. It investigates a population-based sample, using trustworthy instruments, and is pioneering in that it analyzes CVR as measured by the PDAY score in Brazilian adolescents. It also has limitations. Since it is a cross-sectional study, it does not provide a basis for establishing causal relationships between the variables studied and the PDAY risk score – leaving this as an objective for future studies with the goal of early prevention of CVDs.
